# Supplementary figures and images for: An efficient CRISPR-Cas9 enrichment sequencing strategy for characterizing complex and highly duplicated genomic regions. A case study in the Prunus salicina LG3-MYB10 genes cluster
Source: Plant Methods. 2022 Aug 27;18:105. doi: 10.1186/s13007-022-00937-4 (PMC9419362; doi:10.1186/s13007-022-00937-4)

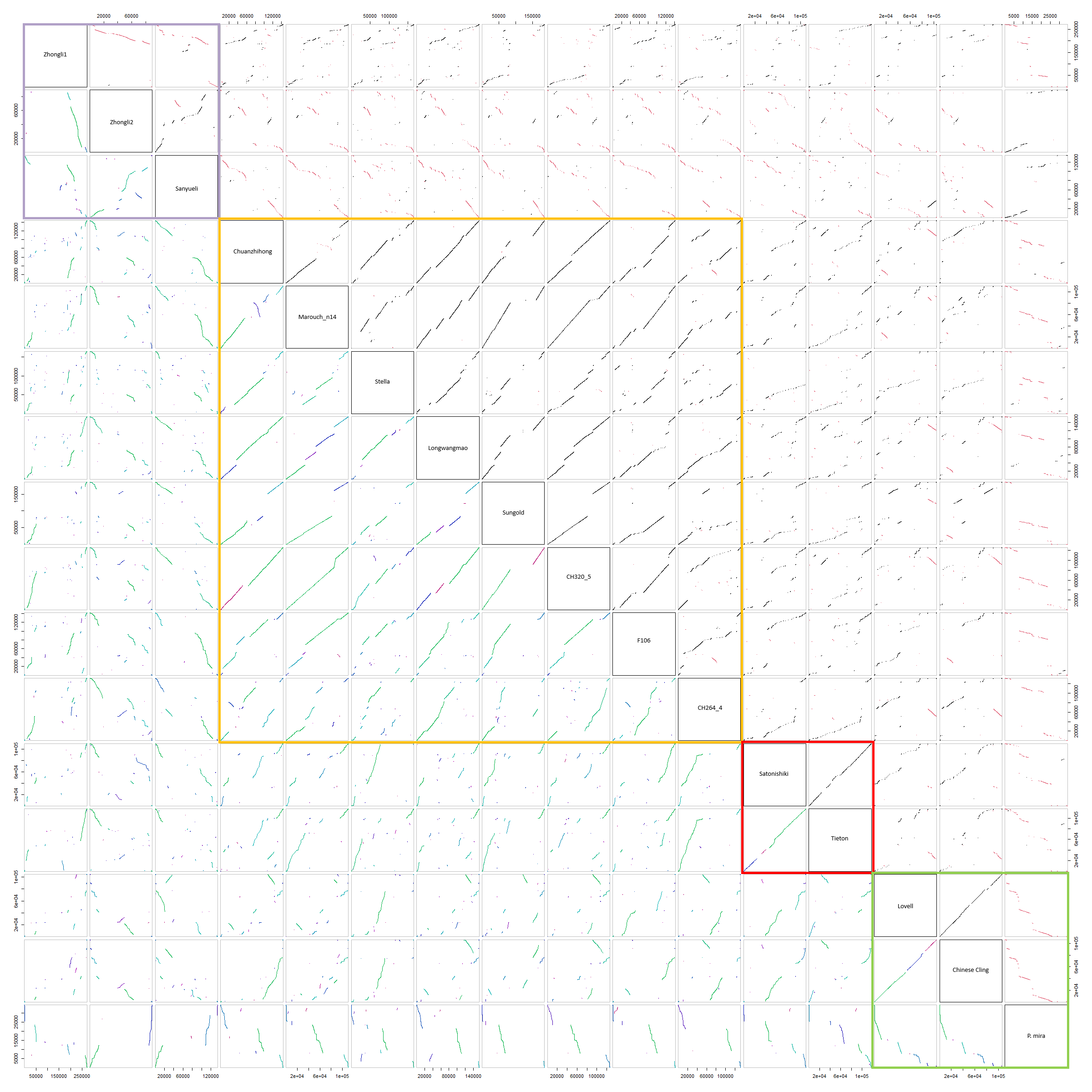

Supplement: Supplementary file 1 — Additional file 1. Dot plot comparing, pair-wise, the MYB10 regions identified in 15 Prunus genomes, represented as in Figure 1a. The colored squares border the Prunus sections considered: purple for Japanese plums, orange for apricots, red for sweet cherries, and green for peaches and its wild relative. [file 13007_2022_937_MOESM1_ESM.tiff]

## Slide 1
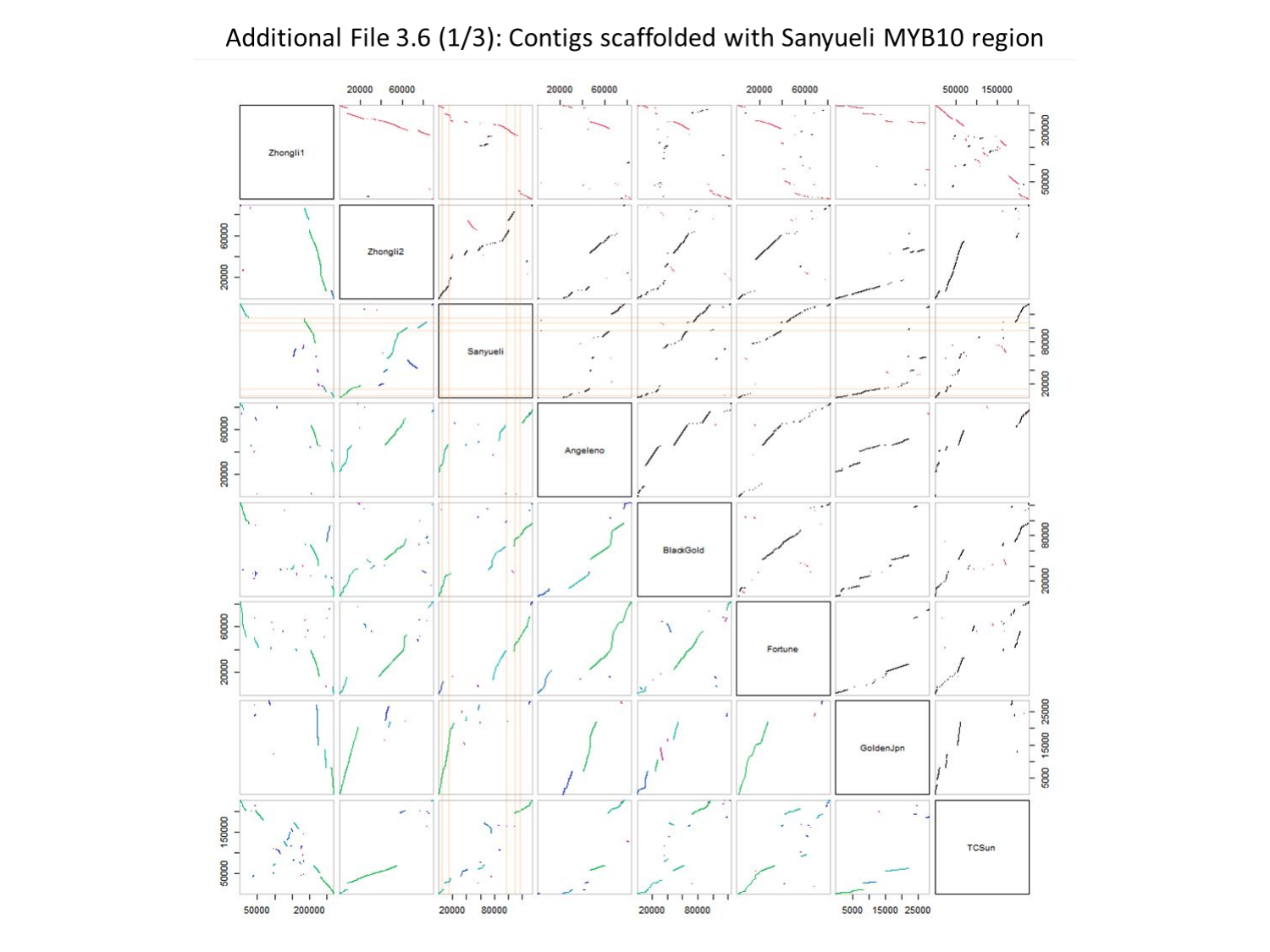

## Slide 2
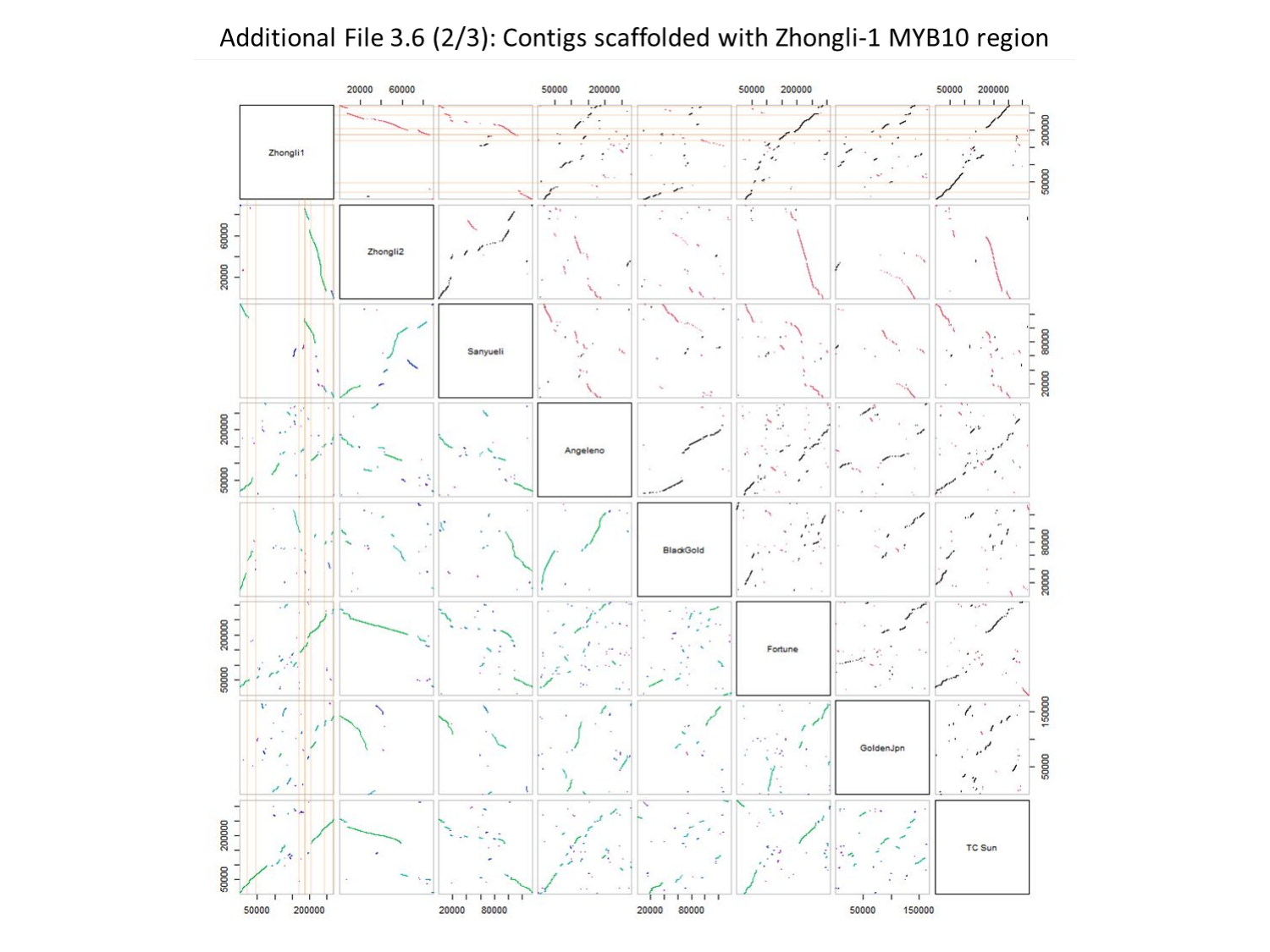

## Slide 3
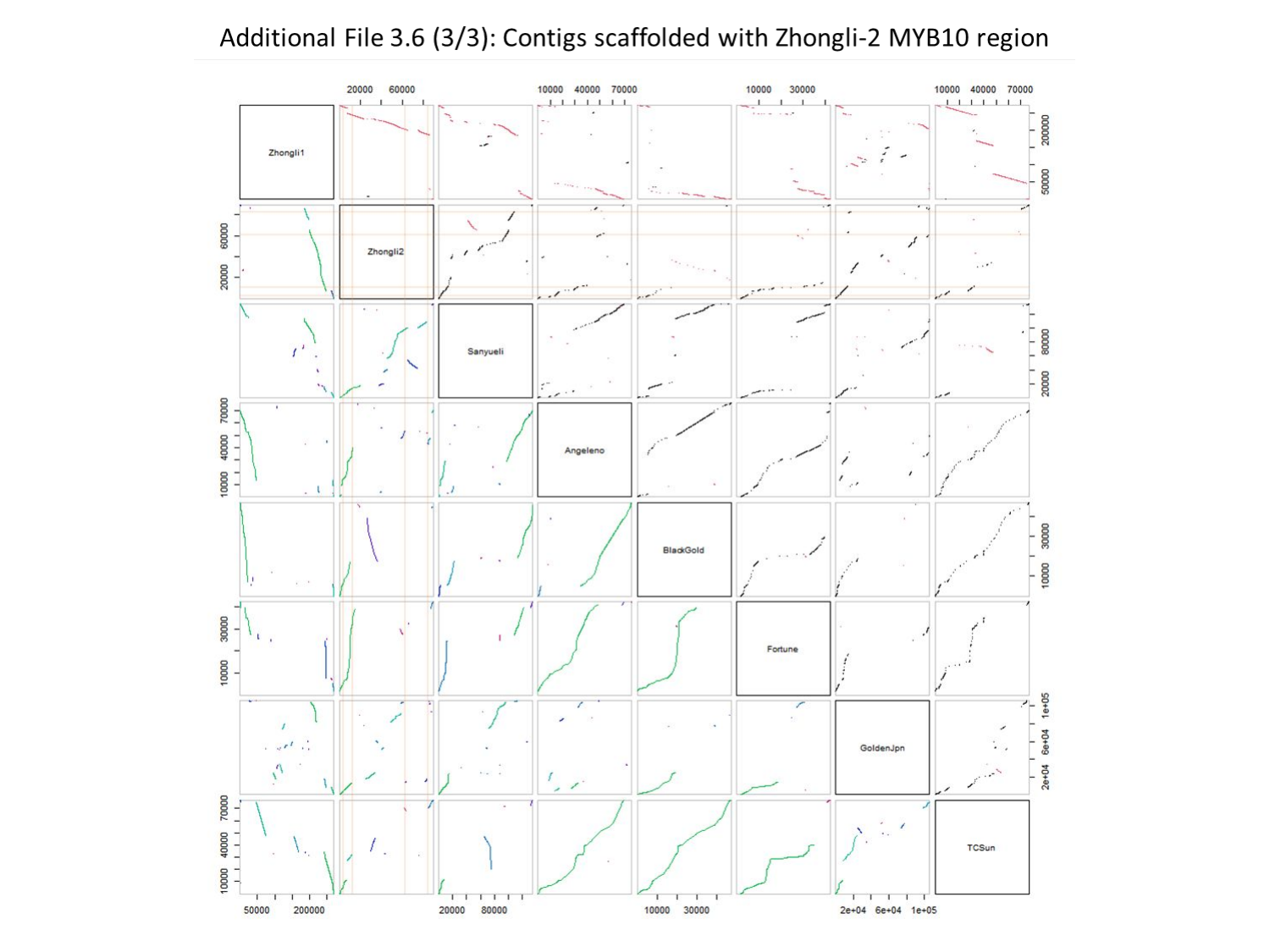

Supplement: Supplementary file 6 — Additional file 6. Pair-wise visualization of the homologous hits (above diagonal) and homologous blocks (below) between each reference region and the de novo contigs scaffolded using the ‘Sanyueli’, Zhongli-1 or Zhongli-2 regions, represented in the same colors as in Figure 1a. The light pink lines show the position of the MYB10 genes in each of the reference regions used for the scaffolding. [file 13007_2022_937_MOESM6_ESM.pptx]

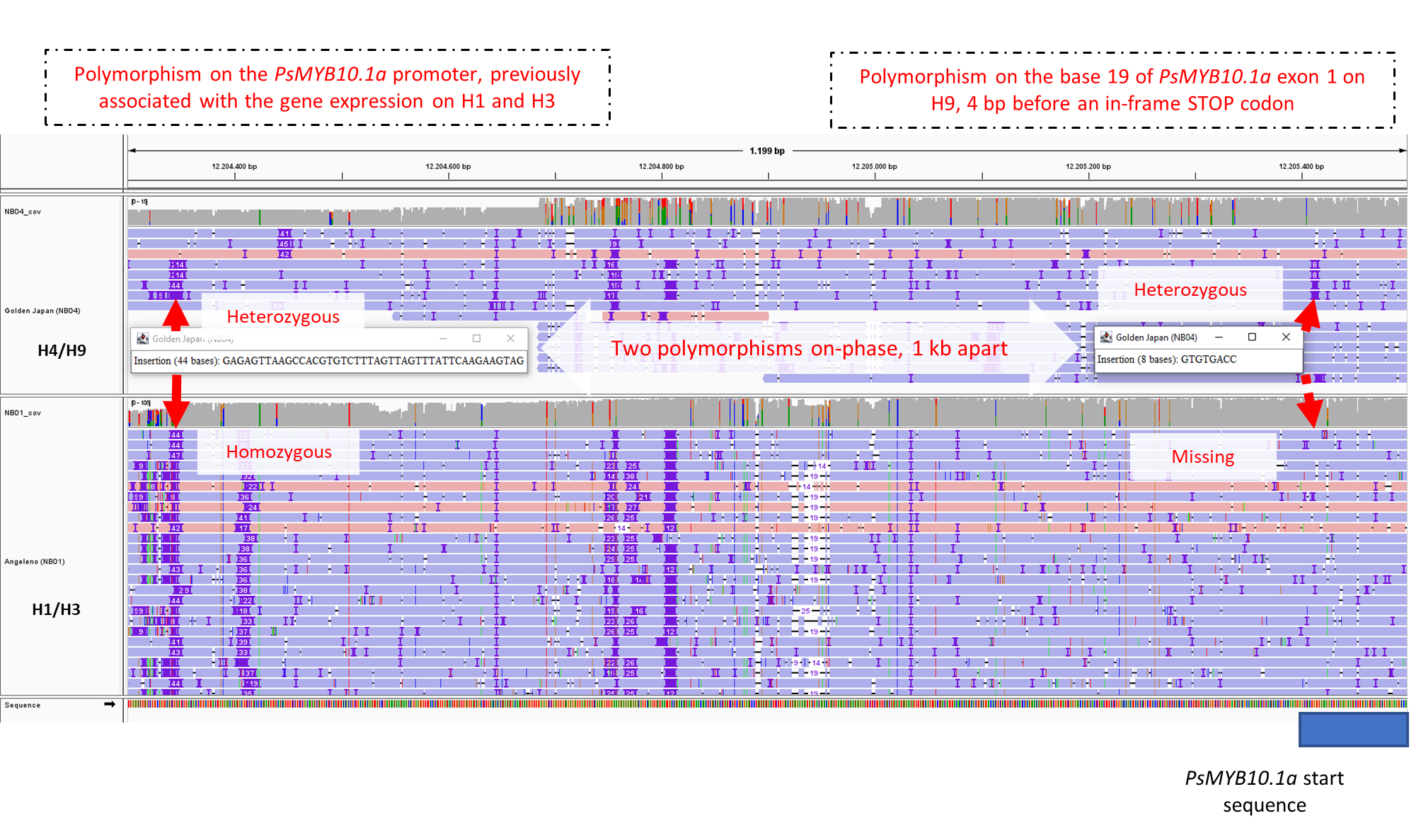

Supplement: Supplementary file 7 — Additional file 7. Visualization of two phased variants 1 kb apart on H9. The 44 bp insertion is present in H1, H3 and H9 and was associated to the red skin color. The polymorphism is phased with an 8 bp insertion at the start of exon 1 of the PsMYB10.1 gene, which explains its lack of function on H9. The reads from H4 do not show either of the two polymorphisms. [file 13007_2022_937_MOESM7_ESM.tif]
