# Supplementary material for: An efficient CRISPR-Cas9 enrichment sequencing strategy for characterizing complex and highly duplicated genomic regions. A case study in the Prunus salicina LG3-MYB10 genes cluster
Source: Plant Methods. 2022 Aug 27;18:105. doi: 10.1186/s13007-022-00937-4 (PMC9419362; doi:10.1186/s13007-022-00937-4)
Supplement: Supplementary file 3 — Additional file 3. Details of the crRNAs designed for the Japanese plum LG3-MYB10 region enrichment. The SNPs identified are underlined and crRNAs were designed including each variant. [file 13007_2022_937_MOESM3_ESM.docx]

**Additional File 3.** Details of the crRNAs designed for Japanese plum LG3-MYB10 region enrichment. The SNPs identified are shown underlined and crRNAs were designed including each variant. The crRNAs and their on-target activity score calculation was obtained using the Alt-R® Custom Cas9 crRNA Design Tool from IDT webpage (https://eu.idtdna.com/site/order/designtool/index/CRISPR_CUSTOM).

| **Name** | **Sequence 5’->3’** | **PAM** | **Target** | **Strand** | **On-target activity score** |
| --- | --- | --- | --- | --- | --- |
| **crRNA-s1** | GGAAGAGCTGTAGACTAAGG | TGG | MYB10.1 and MYB10.2 (exon 2) | (+) | 58 |
| **crRNA-s2** | GGAGGAGCTGTAGACTAAGG | TGG | MYB10.2 (exon 2) | (+) | 50 |
| **crRNA-s3** | GGAAGAGCTGCAGACTACGG | TGG | MYB10.3 (exon 2) | (+) | 57 |
| **crRNA-a1** | ATAAGTCTCTTAGCACCCCT | CGG | MYB10.1 (intron 1) | (-) | 64 |
| **crRNA-a2** | ATAAGTCTCTTAGTACCCCT | CGG | MYB10.1 (intron 1) | (-) | 67 |
| **crRNA-a3** | AAACTTGTCATGAAATTATC | AGG | MYB10.2 (intron 1) | (-) | 51 |
| **crRNA-a4** | ATTGTATAACATCTTTCTCG | AGG | MYB10.3 (intron 1) | (-) | 68 |
| **crRNA-f1** | CGGGTGCAAGGCGTACCAAG | CGG | *Prupe.3G162900* | (-) | 70 |
| **crRNA-f2** | GACTACCCCTGCGAGTCCAG | AGG | *Prupe.3G163400* | (+) | 61 |
