# Supplementary material for: An efficient CRISPR-Cas9 enrichment sequencing strategy for characterizing complex and highly duplicated genomic regions. A case study in the Prunus salicina LG3-MYB10 genes cluster
Source: Plant Methods. 2022 Aug 27;18:105. doi: 10.1186/s13007-022-00937-4 (PMC9419362; doi:10.1186/s13007-022-00937-4)
Supplement: Supplementary file 4 — Additional file 4. Visualization of the depth of the sequences aligned to the ‘Sanyueli’, Zhongli-1 and Zhongli-2 regions. The pink bars highlight the coordinates with previously identified MYB10 gene sequences, where the Cas9 enzyme has two cutting points enabling sequencing in both directions. [file 13007_2022_937_MOESM4_ESM.pptx]

## Slide 1
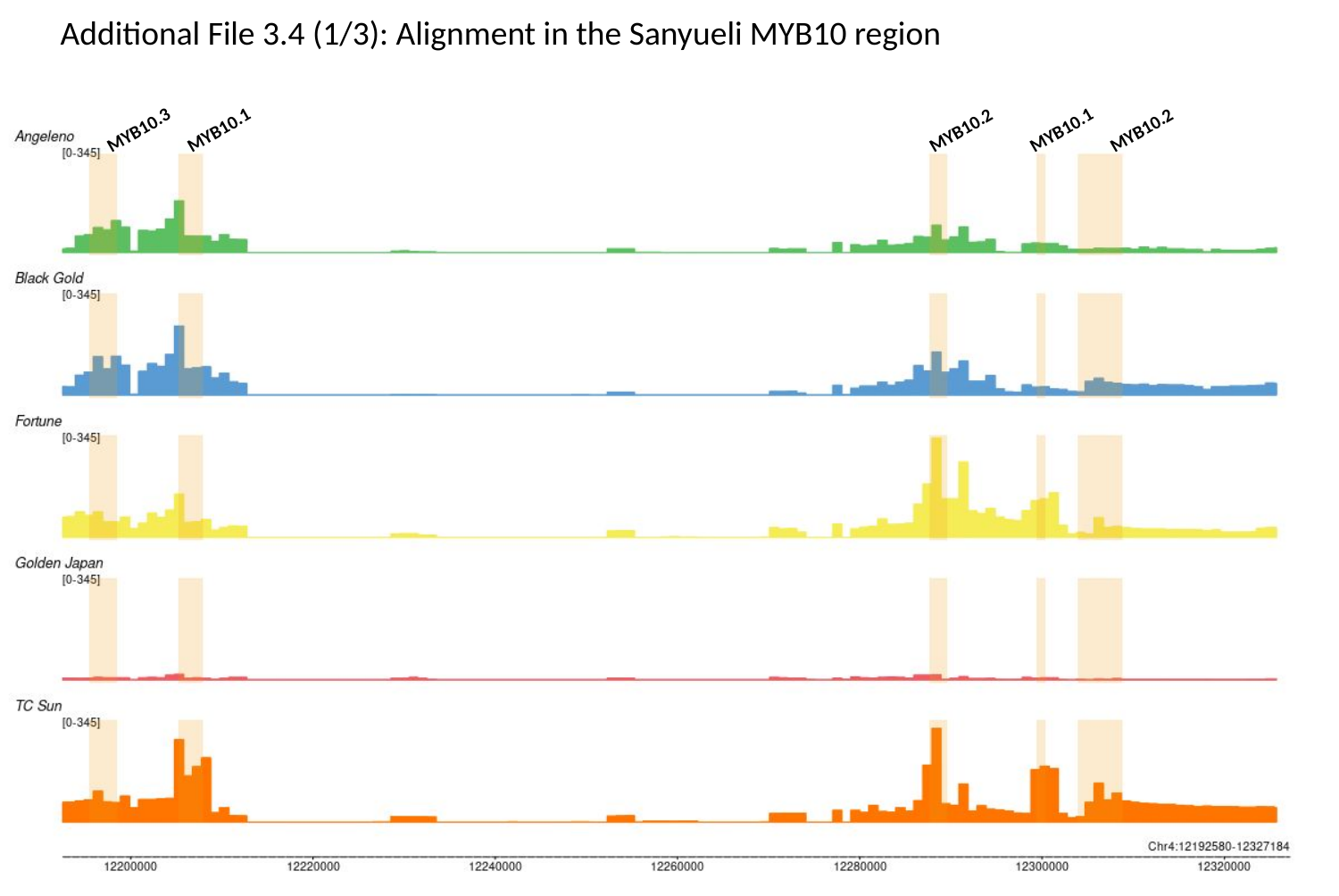

Additional File 3.4 (1/3): Alignment in the Sanyueli MYB10 region
MYB10.3
MYB10.1
MYB10.2
MYB10.1
MYB10.2

## Slide 2
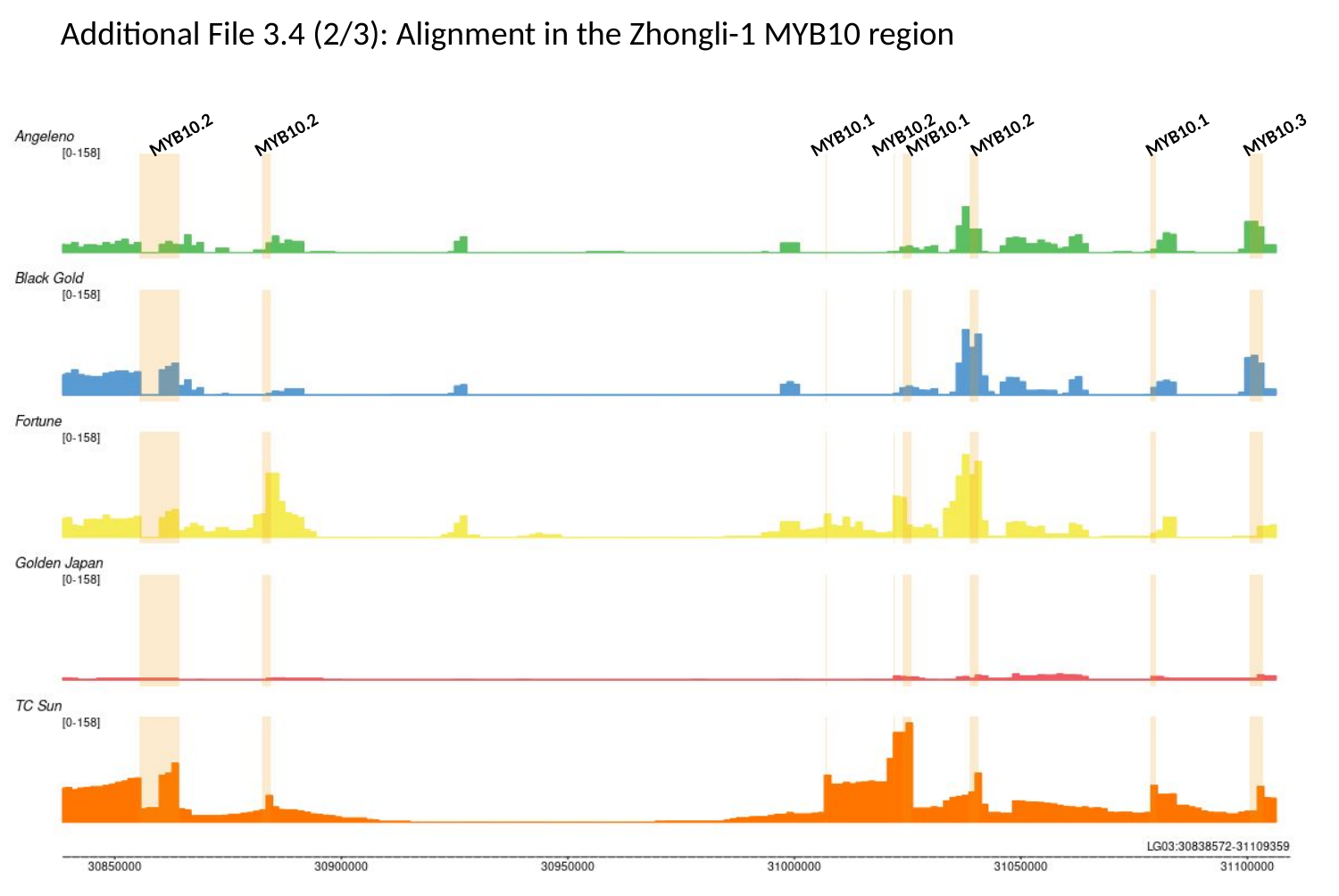

Additional File 3.4 (2/3): Alignment in the Zhongli-1 MYB10 region
MYB10.2
MYB10.2
MYB10.1
MYB10.2
MYB10.1
MYB10.2
MYB10.1
MYB10.3

## Slide 3
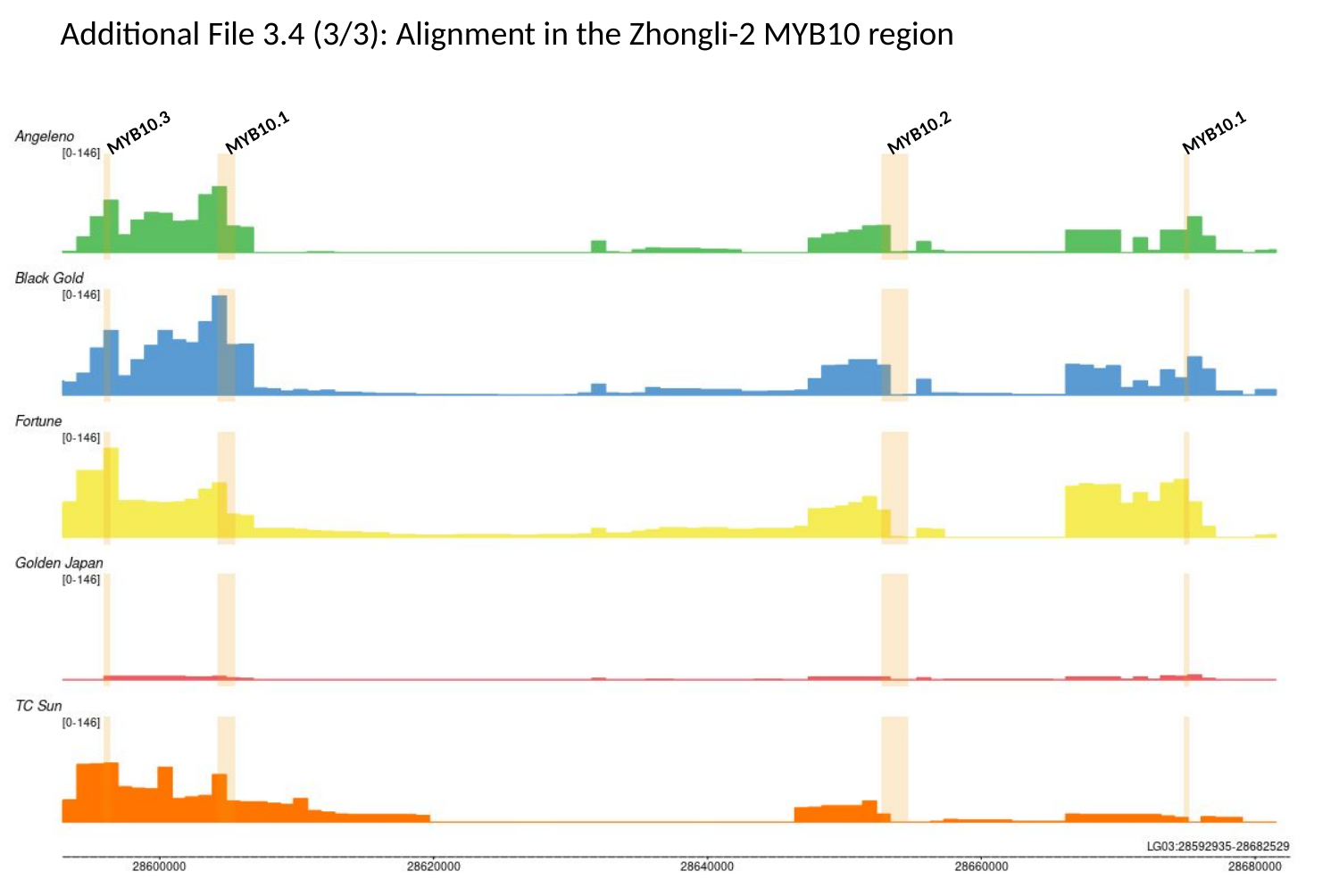

Additional File 3.4 (3/3): Alignment in the Zhongli-2 MYB10 region
MYB10.3
MYB10.1
MYB10.2
MYB10.1
