# Supplementary material for: An efficient CRISPR-Cas9 enrichment sequencing strategy for characterizing complex and highly duplicated genomic regions. A case study in the Prunus salicina LG3-MYB10 genes cluster
Source: Plant Methods. 2022 Aug 27;18:105. doi: 10.1186/s13007-022-00937-4 (PMC9419362; doi:10.1186/s13007-022-00937-4)
Supplement: Supplementary file 8 — Additional file 8. Primer sequences and their annealing temperatures (Ta) used to PCR amplify the MYB10 gene sequences. [file 13007_2022_937_MOESM8_ESM.docx]

| **Primer name** | **Sequence** | **Ta (ºC)** | **Description** |
| --- | --- | --- | --- |
| **M101_RT_F** | TGGACACGAGACATTGCACG | 57 | *PsMYB10.1* amplification (Fiol et al., 2021) |
| **M101_RT_R** | CAATGGTCTTTTGACAGCCGC |  |  |
| **M102_f** | CTGGCTGCAAGCATAC | 57 | *PsMYB10.2* amplification (Fiol et al., 2021) |
| **M102_r** | GTGGGACAAACACTCTC |  |  |
| **M103_f** | ATAGGAACTAGCAGGCAC | 57 | *PsMYB10.3* amplification (Fiol et al., 2021) |
| **M103_r** | AGTTGCTAATAATTGCTACTAGG |  |  |

**Additional File 8.** Primer sequences and their temperatures of annealing (Ta) used to PCR amplify the *MYB10* gene sequences.
